# XperimentR: painless annotation of a biological experiment for the laboratory scientist

**DOI:** 10.1186/1471-2105-14-8

**Published:** 2013-01-16

**Authors:** Chris D Tomlinson, Geraint R Barton, Mark Woodbridge, Sarah A Butcher

**Affiliations:** 1 Centre for Integrated Systems Biology and Bioinformatics, Imperial College London, Sir Ernst Chain Building, South Kensington Campus, London SW7 2AZ, UK

**Keywords:** Experimental annotation, Ontologies, Biological data management

## Abstract

**Background:**

Today’s biological experiments often involve the collaboration of multidisciplinary researchers utilising several high throughput ‘omics platforms. There is a requirement for the details of the experiment to be adequately described using standardised ontologies to enable data preservation, the analysis of the data and to facilitate the export of the data to public repositories. However there are a bewildering number of ontologies, controlled vocabularies, and minimum standards available for use to describe experiments. There is a need for user-friendly software tools to aid laboratory scientists in capturing the experimental information.

**Results:**

A web application called XperimentR has been developed for use by laboratory scientists, consisting of a browser-based interface and server-side components which provide an intuitive platform for capturing and sharing experimental metadata. Information recorded includes details about the biological samples, procedures, protocols, and experimental technologies, all of which can be easily annotated using the appropriate ontologies. Files and raw data can be imported and associated with the biological samples via the interface, from either users’ computers, or commonly used open-source data repositories. Experiments can be shared with other users, and experiments can be exported in the standard ISA-Tab format for deposition in public databases. XperimentR is freely available and can be installed natively or by using a provided pre-configured Virtual Machine. A guest system is also available for trial purposes.

**Conclusion:**

We present a web based software application to aid the laboratory scientist to capture, describe and share details about their experiments.

## Background

The road to ubiquitous and seamless experimental data sharing amongst life science researchers is paved with good intention, yet given the vast effort expended in this endeavour, the reality is still somewhat underwhelming. Many experimental studies now involve the generation of various different modalities of experimental data and use the different views that the data modalities provide to construct and inform mathematical models of biological function. These projects often employ a number of researchers with a wide range of backgrounds and expertise, from the laboratory scientist through service providers to the data analysts and mathematical modellers, who require common language to describe the experiment. In addition to describing the experiment between members of the projects, it is also usually a requirement that any data generated during the lifetime of the project be deposited to the public domain with adequate description to allow the data to be re-used. Minimum information templates, such as MIAME and MIAPE [1,2], and specialised ontologies such as IDOMAL and ENVO [3,4] have been developed to describe and define experiments in fine grained detail. There are a number of software tools currently available to browse and utilise ontologies such as BioPortal [5] and RightField [6]. To a bench biologist working on such a project, this bewildering array of data standards, the accompanying ontologies, the XML and tabular data representations, each with its own confusing acronym, represent a high barrier to entry with seemingly little to gain. It is therefore not surprising that many publicly available experiments still bear minimal annotation despite the commendable community efforts to enforce minimum data standards. One study looking at Affymetrix data in GEO and ArrayExpress identified that only 38% of the microarray data meets the quality and format standards necessary for further integrative analysis [7]. Where publication of an experiment to a public data repository is a condition of the accompanying paper being accepted researchers will generally take the path of least resistance. When experimental data have been extensively annotated and put into the public domain, the (re)annotation has often been added by trained data curators [8], a scarce resource which is well beyond the means of many organisations and research teams.

This paper introduces a software tool that has been designed to address these issues and to assist the bench scientist in describing their experiment in line with appropriate data standards. The result of this effort is a software tool called XperimentR, a rich internet application designed to be used alongside the traditional laboratory notebook, which allows the laboratory scientist to track their experimental procedures and adhere to minimum data specifications with minimal effort.

During our consultation phase, we found much confusion amongst biologists about the use and the role of ontologies in the description of the sample preparation process and of the data resulting from experimental assays. There are some limited examples of the successful use of ontologies to infer information from laboratory data and to combine analysis of experiments from different sources using terms from controlled vocabularies to infer semantic information and to integrate data, which is ultimately the justification of the biologist’s efforts. These examples are the exception rather than the norm and often the problem is exacerbated by the inconsistent use of ontologies both within and between different experiments. The authors feel that this inconsistency stems from a lack of direction to biologists about which ontologies are appropriate in which circumstances. Curators have previously attempted to re-annotate existing data with new (and improved) ontologies or to convert existing annotations between ontologies in a post hoc fashion to achieve data consistency [8].

With these potential problems in mind, XperimentR enables the semi-automatic annotation of experimental data with ontology terms.

## Implementation

### XperimentR design objectives

Taking into consideration the observations outlined in the introduction to this paper, the authors drew up a list of design objectives for a user-friendly experimental annotation tool:

• To minimise the amount of time that the biologist needs to comprehensively describe the sample preparation stages of laboratory experiments

• To simplify and facilitate seamless annotation with ontology entries from appropriate and consistent ontologies

• To enable the experimental information to be stored in a structured format suitable for conversion to the appropriate data standards and for export to the public domain

• To collect and store the experimental information in a secure manner and to allow the user to share the information and data with other users

• To be universally available to the user through the internet without requiring any complex installation process for novice users

### XperimentR software architecture

XperimentR is a web based software application developed using Adobe Flex: an open source Rich Internet Application framework. Flex enables familiar user interface components to be embedded in a standard web page whilst giving the programmer access to an extensive library of graphical user components. The Flex environment gives the developer the convenience of web distribution via any Flash enabled internet browser combined with the ability to create interactive and responsive software with a complex graphical user interface. As the future lifetime of Flash is now limited due to technological advances, it is highly probable that any future incarnations of XperimentR will be implemented in HTML5 and javascript.

To facilitate fast-paced and intuitive experimental annotation a central feature of XperimentR is the representation of the experimental process as a graph, a conceptualisation first introduced by ArrayExpress [9]. The Flex environment facilitates the seamless inclusion of interactive graphical components by the user, such as the graph component and its nodes and arcs (also known as links or edges). Nodes of the experiment graph represent physical laboratory entities, such as biological sources (Human, Mouse, Bacteria etc), laboratory containers (Eppendorf tubes, flasks, fermenters etc) and experimental assays including microarrays, microscopy images and mass spectrometry data. The arcs of the graph represent actions (or transformations) linking nodes of the graph, usually laboratory protocols.

## Results and discussion

### XperimentR user interface design

**Figure 1:**
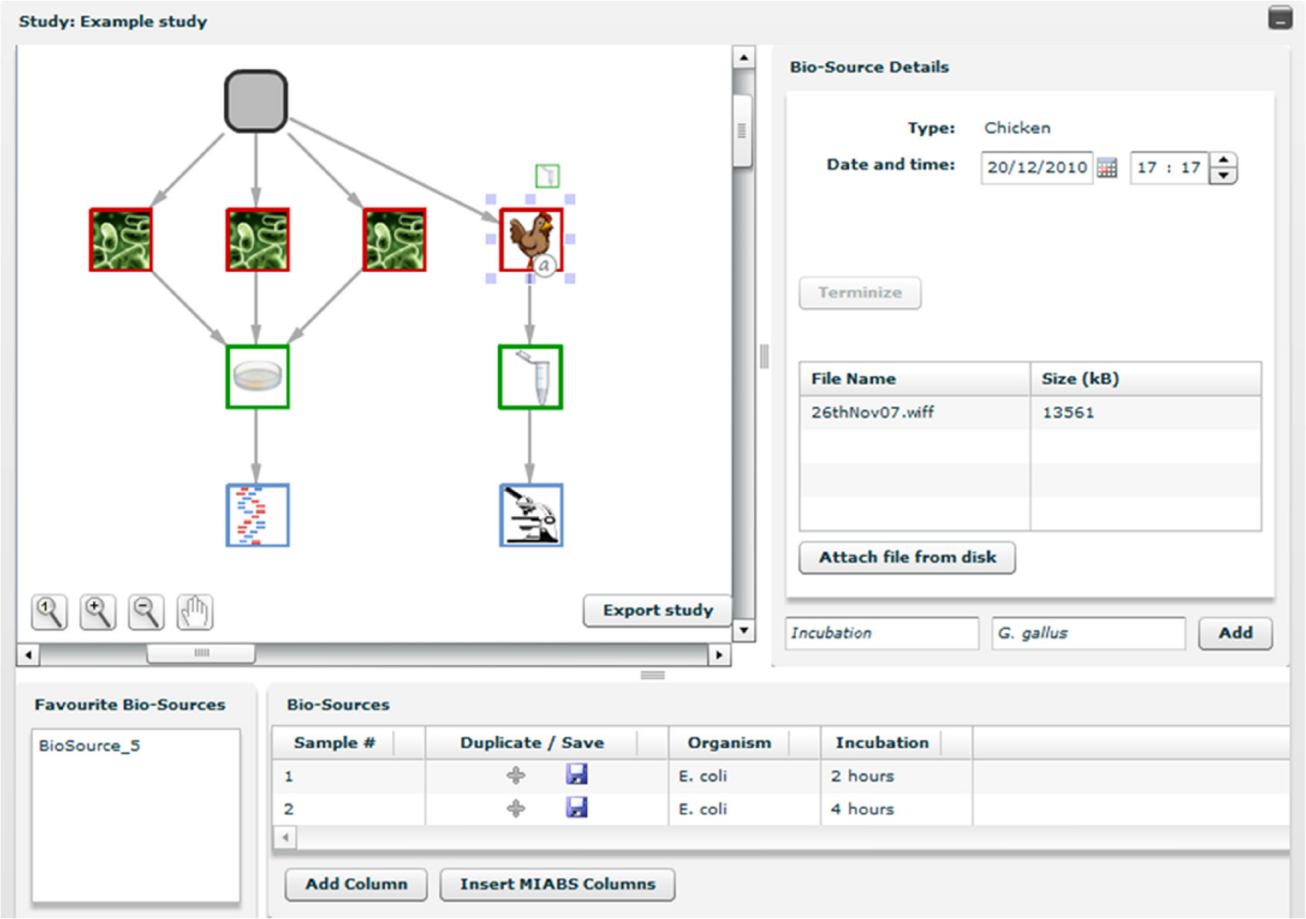
**The XperimentR study interface showing the study graph. **The study graph is in the central left panel of the study interface. This example shows a simple study with four biosource materials (one chicken and three bacterial icons). These are shown in the second row of the graph (the root node represents the study as a whole). Source materials are extracted to the petri dishes and eppendorf tube in row three (the viral biological replicate samples being pooled). Finally these are deposited in the assays on the final row of the graph. The assays in this example are sequencing and imaging.

The XperimentR user interface was designed to enable the rapid, detailed annotation of experimental processes and procedures. The central feature of the interface is the Study graph as shown in Figure 1. The graph represents the experimental process as a whole and is laid out as a tree, with its root node representing the Study (or experiment) as a whole. Every XperimentR graph has a single, unique root node as it represents a single unique Study. Other than the root node, the graph can contain only three types of nodes; biosources, containers and assays. Arcs in the graph always represent actions taken by the laboratory biologist (with the exception of those connected to the root node, which represent the inclusion of biosources in the study). The second level nodes represent the biosource, as a biological laboratory experiment will (almost) always involve the use of some raw biological materials as input. A biosource node must have as its child (or children), a container node with the arc between them representing the action of extracting a biomaterial from the biosource and putting it into a container. A container can have as its child, another container or a biological assay with the arc between them typically representing an experimental protocol as the sample gets processed.

**Figure 2:**
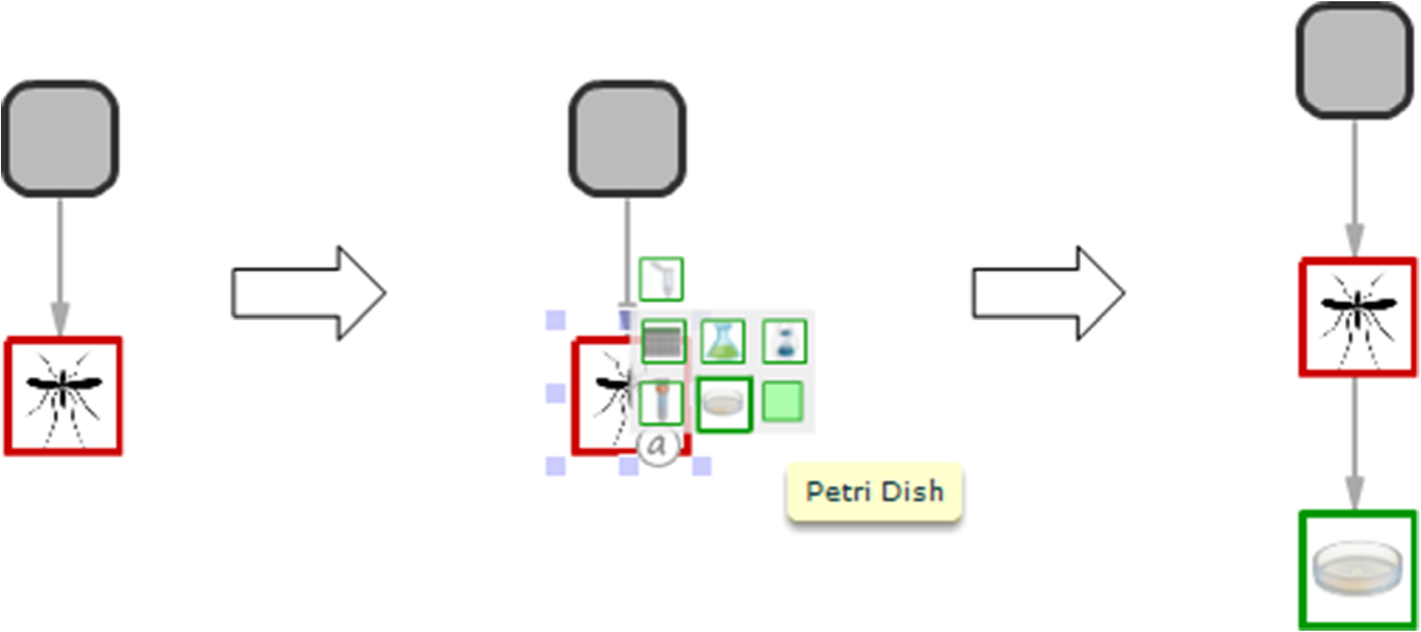
**The XperimentR popup menu when clicking on a Biosource. **When the user clicks on a node in the graph a popup menu appears. The items in the popup menu are constrained by the type of node that is being selected (biosource, container or assay). As the user moves the mouse over the icons a popup will display notifying the user of the name. To add an item the user simply drags the item from the popup menu to the graph canvas.

The user can zoom in and out of regions of the graph using the controls embedded in the lower left of the graph panel thus enabling larger experiments to be navigated and annotated with ease. To add a node to the XperimentR graph the user simply clicks on the parent node and a popup menu will appear as shown in Figure 2. The menu shows the available options (which are constrained by the type of the parent node) as small icons. As the user moves the mouse over the small icons a tooltip shows the type of entity that the user has moused over. The user can then drag the correct node type onto the graph canvas and a connecting arc to the parent is also added.

Annotation to any node or arc in the graph can be performed by clicking on it and using the upper right panel as shown in Figure 1. Name and value pairs can be added to all graph nodes as annotation and arbitrary files can be added to any node or arc of the graph. This feature allows the biologist to attach notes, output and parameter files to the annotated information. Protocol applications are added by clicking on the arc of a graph and attaching the appropriate protocol by choosing from a list via the protocol application panel on the right. There is a field in the protocol application panel (action) to record any deviations from the experimental protocol or unusual circumstances.

When the construction and annotation of a study graph is complete, the user may add ontology terms to the annotation by using the Terminize feature. Terminize traverses the study graph, using the Ontology web service to check annotation and text fields, presenting the user with any Ontology terms it returns. The user can then select the most appropriate to be attached to the annotation.

Samples can be split or pooled. A user of XperimentR is able to interactively edit the experimental graph by adding, deleting and editing the nodes and arcs using the mouse and the various features of the application. Users can edit the annotations of several graph nodes (or arcs) at the same time and duplicate and store various entities and configurations.

### XperimentR user interface short cuts

XperimentR is designed to aid the quick and painless annotation of laboratory experiments. As identical laboratory procedures are often repeated on different biological samples, which may also have the same attributes, we have designed several timesaving features as a part of XperimentR.

Users can duplicate biosource objects and all of the associated annotation. To action this, a user simply presses the appropriate + button in the biosource table and the biosource will be duplicated.

XperimentR allows users to apply annotation to several objects at once by allowing multiple selection of objects in the Study graph. This feature is only available for graph objects of the same type. A user can achieve this by either individually selecting the items or by drawing a rectangle around the graph items. The user may then alter the properties of all of the selected items by editing the details panel on the upper right hand side. In a similar way, multiple child objects can be added to the Study graph simultaneously. To action this a user selects a group of parent objects and then adds a child node to one of them – child nodes of the same type will be added to all selected nodes. All of these features markedly speed up the annotation process as similar or identical processes are commonly applied to different laboratory samples that have identical (or very similar) properties.

### Server side

To enable the rapid development of the backend of the system the authors selected the Omixed [10] system to build and interface to the XperimentR data store. Omixed is a model driven biosciences data management system with a built in user access control mechanism. In Omixed, the developer first can specify an XML data model and use the Omixed builder to build the underlying data store (in this case a PostgreSQL database). The data store is then accessed via calls to an XML web service provided by a Java Servlet. Omixed also provides various client language interfaces to the data store web service interface. The Omixed Flex and PHP libraries are used to arbitrate the data store communication in XperimentR. Omixed has user access control built into it at the object level and this mechanism is used to facilitate data privacy and sharing of XperimentR data items.

### The XperimentR data model

The XperimentR data model is based on the ISA-Tab [11] (Investigation, Study, Assay) data standard which was designed to allow the annotation and sharing of experiments involving data from several different experimental modalities. ISA-Tab is essentially a modality neutral dialect of MAGE-Tab [12], an established standard for microarray data, which itself is related to an XML representation of microarray data called MAGE-ML [13]. MAGE-ML was the original data transport format for the MAGE object model [14] representation of microarray data and therefore the XperimentR object model bears some similarity to the MAGE object model.

The decision to adopt this ISA-Tab / MAGE approach was taken as it met the design criteria identified in our preliminary meetings with biologists in that the concepts and entities identified in the ISA-Tab and MAGE standards coincide with the important concepts and entities within the biologist’s view of the experimental process. XperimentR is designed to allow the annotation of experiments from any (or many) modality(ies), thus also fitting in with the ISA-Tab rationale. The root item in the XperimentR data model is the Investigation, a container for a collection of Studies. A Study, the ISA-Tab equivalent of a laboratory experiment, can contain zero, one or more experimental Assays (which do not have to be of the same experimental modality). Other important items in the data model include Biosource, Biosample (equivalent to a laboratory container), Action, Protocol and OntologyTerm. The data model is expressed in XML format and this is used to build the entities in the data store. The full XML data model and the associated entity relationship diagram are contained in Appendix I of this paper.

### Minimum information about a biological sample and technology templates

There are a number of details concerning the biological sample which should be captured regardless of the technology being used to generate the data. This includes for example the species, strain and genetic modification. In XperimentR these details are called MIABS (Minimum Information About a Biological Sample). XperimentR can also be used to capture the minimum information about the technology used to generate the data. Users can create a template for example to capture information about a microarray technology. The user can then save a completed template for a particular technology, such as an Affymetrix Hgu133 array. They can then associate the completed template with multiple data files with a single mouse click. The MIABS data can then be compiled together with the appropriate details from the specific technology template to conform to minimum information standards.

### XperimentR export

The data model described in the previous section can be used to enable the construction of data output adapters for data export and reporting. So far three output adapters have been built for XperimentR; an ISA-Tab exporter primarily designed for data sharing and publication, a PDF document giving protocol information (for laboratory use) and a PDF representation of a study and the items and actions used in it (for paper or file based recording of experimental processes).

The protocol output information is achieved by using the Omixed Java interface and the use of a servlet to construct and output the PDF document. The ISA-Tab and Study reporter have been built using the Omixed PHP interface. Data about a study or Investigation from the Omixed data store is read via the Omixed PHP web service interface into a PHP data model. The PHP model can then be traversed and relevant data output and sent to the user in the format required. This approach is extensible in that new adapters for other data output formats can be built very cheaply using the existing PHP data model which accurately reflects the stored data and its structure. ISA-Tab documents constructed from XperimentR have been checked using the ISAValidator tool [15] and found to conform to the ISA-Tab standard and therefore can be uploaded to the Bio Investigation Index [16] data repository for publication.

### XperimentR ontology lookup service

Users can annotate the terms and descriptions used to describe the nodes and arcs with ontology terms. A set of pre-determined ontologies is set up for each Investigation to enable the consistent and compatible annotation both within and across Studies.

XperimentR takes advantage of web services to lookup ontology terms based on the sample annotation entered by the user. In effect a text string is sent to the service, along with the selected ontologies to be searched, and the service returns ontology terms that match the syntactic or semantic content of the string. Ontology terms that match are presented to the user who can then select the terms to be associated with the text string and referenced in the XperimentR data store.

In developing this feature, we first looked at the existing ontology web services available, namely OLS [17], BioPortal [5] and Terminizer [18] and integrated first Terminizer and then OLS into XperimentR. Upon experimentation with these resources, the authors found that neither of the services fully matched the requirements of XperimentR. The Terminizer service only performed full text matching for search strings against a predefined ontology set and OLS lookups suffered from variable and significant latency. Neither allowed the addition of supplementary ontologies. Therefore we built our own lightweight Ontology lookup service specifically to meet the needs of XperimentR. The XperimentR ontology service provides a simple search service and can be populated with ontologies in OBO format. Ontology information is returned via a web service interface in JSON format. This component is completely decoupled from the XperimentR client application and can be used as a standalone service by other applications.

### XperimentR protocol use and management

When meeting with laboratory biologists to discuss the important features of XperimentR, it quickly became apparent that the inclusion and management of laboratory protocols was a very high priority for those working with biological samples. XperimentR endeavours to simplify the handling of protocols by providing a simple interface for the user to input and search laboratory protocols. Salient points are;

• Once entered into the XperimentR system the text of a protocol may not be altered. If a user deviates from the defined steps then there is a provision to record the deviations.

• Protocols can be viewed, selected and downloaded via a searchable web interface.

• Protocols can kept private or be shared with other users of the system

• A user can attach arbitrary comments to a protocol. These are also searchable along with the text of a protocol.

Protocols are added via a user interface and are included in experiments by attaching them to actions (represented by the arcs of the study graph). An output adapter exists for individual protocols where the information can be downloaded as a PDF document.

### XperimentR data security

XperimentR handles security at the individual object level via the built in Omixed security layer. The Omixed security layer makes each object that is created by a user (such as a biosource, container or action) automatically private and only accessible and editable by its creator. Objects can be made more widely available by adding an access group to the object’s permissions. Individual users and groups of users may be added to any access group. Access groups can be given read only permissions, or full read, write and delete permissions.

XperimentR builds on this infrastructure by giving each Investigation a unique access group when it is created. The creator of an investigation can then add and remove other users from the investigation’s access group and also give the individual users read only access or full access to the data items within an investigation.

XperimentR will automatically log a user out of the system if there is no user action in an XperimentR session for more than 10 minutes.

### XperimentR integration with underlying data repositories

**Figure 3:**
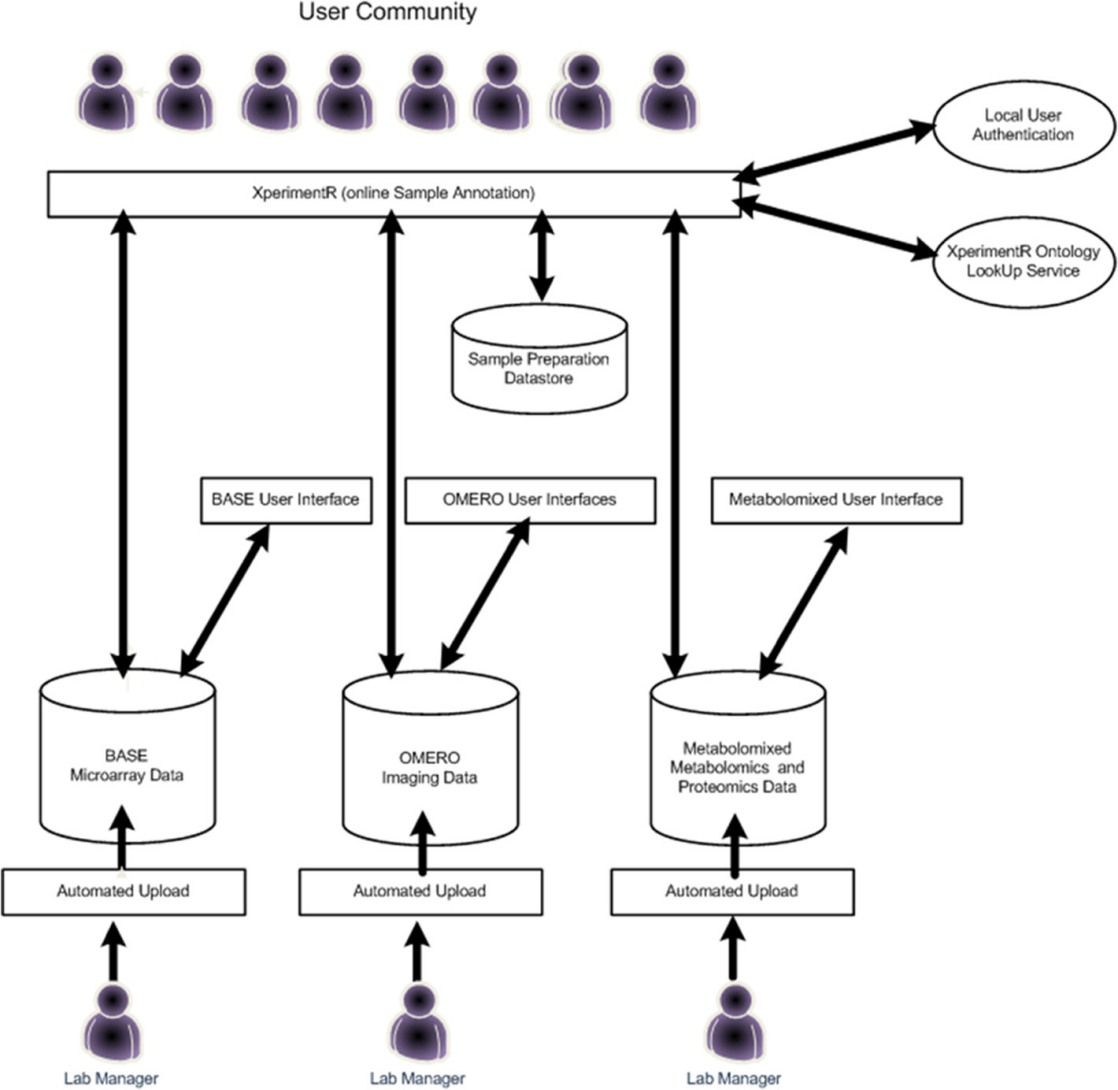
**XperimentR system architecture at imperial college. **The diagram shows how XperimentR fits into the biological data management architecture at Imperial College. It is integrated with three data repositories that can store different types of biological data.

**Figure 4:**
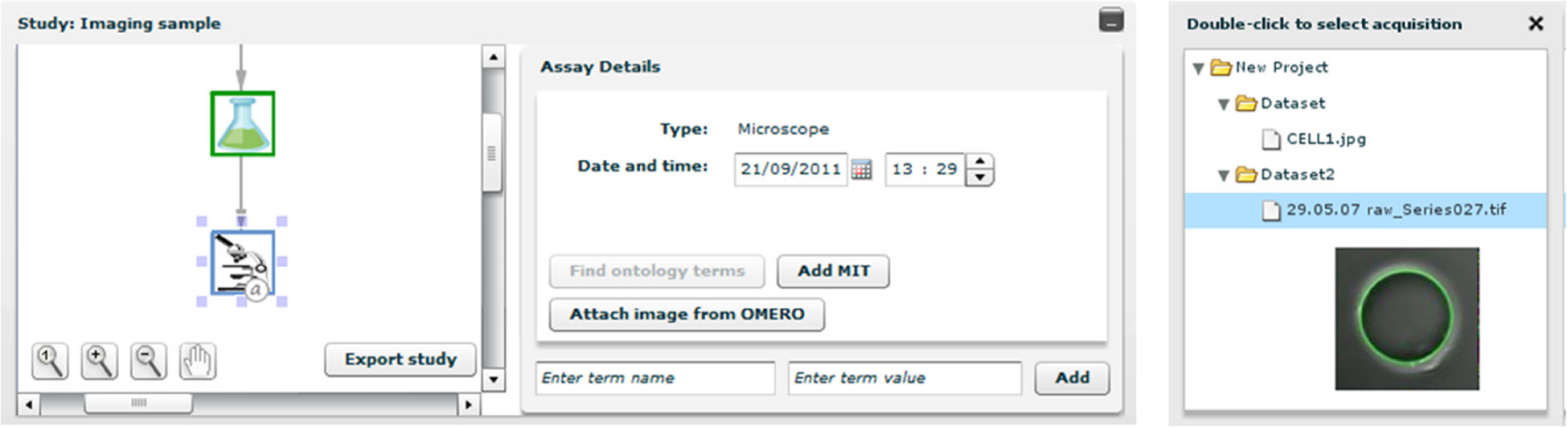
**The XperimentR / OMERO integration user interface. **Screenshot showing XperimentR integration with OMERO. The user can attach an image stored in OMERO to an XperimentR assay graph node (for microscopy). The user selects the required image via the popup window which queries the OMERO data store using the user credentials of the current XperimentR user. The images that the user has access to are displayed in a directory tree with levels representing Projects, Data Sets and Images. As the user moves the mouse pointer over the Image nodes of the tree thumbnail versions of the image are rendered in OMERO as shown. The user can link the image to XperimentR by clicking on it. The image and metadata is then available for download through XperimentR.

XperimentR can be used as a standalone tool or can be integrated with underlying data repositories. The current configuration of the biological data management architecture at Imperial College is shown in Figure 3. As shown, the Imperial installation of XperimentR is currently integrated with three underlying data repositories which store raw data and assay metadata from different experimental modalities. These are BASE [19] for transcriptomic data, OMERO [20] for Imaging data and an in-house data store called Metabolomixed that can store data from MS and NMR equipment, thus covering both the Proteomic and Metabolomics experimental modalities. Essentially the integration takes the form of a link between an XperimentR assay object and the corresponding experimental data object in the underlying data store. Customised code is then written in the XperimentR interface to retrieve and display (or download) data files and assay metadata from the underlying data repository. The form of this code depends on the API available for the underlying store. Where ever possible this link is made seamless to the user and no extra user authentication is required. As an example, Figure 4 shows the OMERO integrated user interface.

## Conclusion

There is a clear need to recognise the importance of the detailed annotation of biological experiments and of adherence to the existing data standards for recording experimental information to ensure the future proofing of data generated from today’s experiments. We also recognise that the laboratory biologist will have a different set of priorities and will personally gain very little from the time consuming process of detailed experimental annotation. In developing XperimentR, we have put the needs of the laboratory biologist at the centre of the annotation process and developed a tool which can be used painlessly alongside a laboratory notebook to record the sample preparation steps taken in the laboratory. The tool is user friendly, has several time-saving enhancements and is standards compliant. Our focus, in this endeavour, has been on the user experience and how best to capture the relevant information without distracting the scientist from the tasks within the laboratory.

## Availability and requirements

• Project name: XperimentR

• Project home page: http://www3.imperial.ac.uk/bioinfsupport/resources/data_management/xperimentr

• Operating system(s): Platform independent

• Programming language: Java, PHP, Flex

• Other requirements: PostgreSQL

• License: GNU General Public License v3.0

• Any restrictions to use by non-academics: no limitations

XperimentR is freely available from the project website and has no usage restrictions. It is implemented using Java, PHP and Flex and tested on CentOS Linux 6. It requires the Apache HTTP Server, the PostgreSQL database and Apache Tomcat. Optional dependencies are the OMERO image management system ([20] - http://www.openmicroscopy.org/site), the BASE microarray database ([19] - http://base.thep.lu.se/) and Metabolomixed NMR/MS storage system (more information available on request).

## Competing interests

The authors declare that they have no competing interests.

## Authors’ contributions

CT responsible for the initial data model design, designing and implementing XperimentR output adapters, some modifications and improvements to XperimentR interface, overall project management. GB conceived XperimentR and implemented several user interface features, liaison with biologists, produced first fully functional XperimentR prototype. MW development of release candidate from XperimentR prototype, ontology service and data silo connectors, construction of XperimentR virtual machine and deployment of production and demonstration systems. SB secured funding for this work and oversaw all aspects of the project. All authors read and approved the final manuscript. 
